# Multi-trait genomic-enabled prediction enhances accuracy in multi-year wheat breeding trials

**DOI:** 10.1093/g3journal/jkab270

**Published:** 2021-07-30

**Authors:** Abelardo Montesinos-López, Daniel E Runcie, Maria Itria Ibba, Paulino Pérez-Rodríguez, Osval A Montesinos-López, Leonardo A Crespo, Alison R Bentley, José Crossa

**Affiliations:** 1 Departamento de Matemáticas, Centro Universitario de Ciencias Exactas e Ingenierías (CUCEI), Universidad de Guadalajara, Guadalajara 44430, Mexico; 2 Department of Plant Sciences, College of Agricultural & Environmental Sciences, University of California Davis, Davis CA 95616, USA; 3 International Maize and Wheat Improvement Center (CIMMYT), Carretera México-Veracruz, México; 4 Colegio de Postgraduados (COLPOS), Montecillos, Edo. de México, México; 5 Facultad de Telemática, Universidad de Colima, Colima, México

**Keywords:** wheat, wheat quality, multi-trait analysis, multi-environment analysis, genomic prediction, GenPred, shared data resource

## Abstract

Implementing genomic-based prediction models in genomic selection requires an understanding of the measures for evaluating prediction accuracy from different models and methods using multi-trait data. In this study, we compared prediction accuracy using six large multi-trait wheat data sets (quality and grain yield). The data were used to predict 1 year (testing) from the previous year (training) to assess prediction accuracy using four different prediction models. The results indicated that the conventional Pearson’s correlation between observed and predicted values underestimated the true correlation value, whereas the corrected Pearson’s correlation calculated by fitting a bivariate model was higher than the division of the Pearson’s correlation by the squared root of the heritability across traits, by 2.53–11.46%. Across the datasets, the corrected Pearson’s correlation was higher than the uncorrected by 5.80–14.01%. Overall, we found that for grain yield the prediction performance was highest using a multi-trait compared to a single-trait model. The higher the absolute genetic correlation between traits the greater the benefits of multi-trait models for increasing the genomic-enabled prediction accuracy of traits.

## Introduction

Wheat is one of the most important cultivated crops in the world and is a major source of energy and protein in the human diet. It is used to produce a diversity of foods with specific end-use requirements including protein quantity, quality, and kernel hardness (Peña *et al.* 2002). Wheat consumption has grown continuously, leading to a gradual increase in the industrial production of wheat-based foods (Shewry *et al.* 2003; Shewry and Hey 2015) with strict and uniform quality requirements for processing. 

Genomic prediction (GP) uses the phenotypic and genotypic data of a training population to predict the phenotypic values of a test population that has only been genotyped. In the context of wheat breeding, a major objective is selection of lines with high grain yield performance and good grain quality. Initial breeding cycles select on grain yield performance, while quality traits are improved in later stages due to the scale of seed requirements and the financial and time cost of quality assessments. GP could improve selection accuracy in both early and later breeding stages by improving the overall grain yield of lines in the first stage and considerably reducing the cost of the screening process in the second multi-trait selection stage (Ibba *et al.* 2020).

Considerable research has been done in recent years to improve the prediction accuracy of GP models aimed at developing single-trait models and, more recently, multi-trait models (*e.g.*, multivariate analyses). Single-trait models are trained to predict the value of a single continuous (or categorical) phenotype in a testing data set, while multi-trait models are trained to predict two or more traits simultaneously. The extension from single-trait to multi-trait linear mixed models that estimate and use trait correlations to calculate best linear unbiased predictions (BLUPs) of genetic value is well established (Henderson and Quaas 1976). In general, multi-trait models represent complex relationships between traits more efficiently as they not only exploit correlations between lines, but also correlations between traits. The genetic correlation between traits is the basis for the benefit of a multivariate analysis for GP as the higher the absolute genetic correlation between traits, the greater the benefit of the multivariate analysis. Parameter estimates have greater precision accounting for the genetic (and residual) correlation between traits and environments under study (Montesinos-López *et al.*^2016^, 2019a,b). In addition, multi-trait models can improve indirect selection, since they increase the precision of genetic correlation parameter estimates between traits (Montesinos-López *et al.* 2016, 2018a,b).

The wheat multi-trait quality information produced in each breeding cycle can be used to develop GP models that may help reduce the number of lines for quality trait analyses. Battenfield *et al.* (2016) showed that for quality traits in wheat, a higher prediction accuracy was obtained when the size of the training population increased over years. Ibba *et al.* (2020) have shown moderate to high genomic-enabled prediction performance of wheat quality traits in consecutive years.

Assessing the effectiveness of genomic-enabled prediction should measure how accurate the prediction of the genetic values ($\hat{g}$) is in comparison with the true unknown and unobservable genetic value (*g*). The predictive ability [the correlation between the observed phenotypic value, *y*, and genomic estimate breeding value GEBV ($\hat{g})]$ of the different GP models is usually used (Dekkers 2007). Different random cross-validations (CVs) schemes are proposed to measure these predictive abilities for single-trait analyses. Burgueño *et al.* (2012) and Jarquín *et al.* (2014) studied the prediction ability of genotype and genotype × environment interaction (G × E) for single-trait models using two random CV schemes, one that evaluated the prediction of a proportion of lines unobserved in all the environments (CV1), and another that evaluated the predictions of lines observed in some environments but not in others (CV2).

However, since true breeding value (g) is unknown, the Pearson’s correlation between the observed phenotypic value, *y*, and the genomic estimate breeding value GEBV, $\hat{g},$ overestimates the true prediction accuracy. An estimate of the true accuracy can be obtained by dividing the correlation of $\hat{g}$ and *y* by $\sqrt{h^{2}}$ (where $h^{2}$ is the heritability of the trait). This correction of Pearson’s correlation attempts to account for the unknown value of g and works well if the estimates of variance components are reasonable and precise. However, accuracy in the estimation of heritability is of paramount importance, since it might cause artificial increases or decreases in the reported prediction accuracy of genomic-enabled predictions, and this could have implications for the evaluation of multi-trait GP models. Other criteria commonly used to select the best predictive models are the mean squared error prediction (MSEP) and the mean arctangent absolute percentage error (MAAPE) (Ibba *et al.* 2020), which also have the same problem as the Pearson’s correlation when used to evaluate the performance (prediction accuracy) of the true genetic, because they also are based on the observed phenotypic values.

As an alternative to correcting the Pearson’s correlation by $\sqrt{h^{2}}$, Runcie and Cheng (2019) derived additional methods to pre-correct the correlation between the observed and predictive values. The aim was to predict the performance of one trait using information from other traits in other individuals, similar to the CV2 case described above. They observed that there is bias in estimates of GP accuracy when there is an exchange of nongenetic information between traits (or environments or individuals) through the model; this only happens in random CV2 and depends on the covariance parameters between traits. Intuitively, there is dependency on the predictions of the same individuals with secondary traits and, in general, the observations are not independent, as they have a covariance structure intra-traits and between traits. The Runcie and Cheng (2019) results are promising and the authors concluded that precautions must be taken when CV schemes are applied to multi-trait predictions to avoid biased results when secondary traits are used to predict primary traits.

Ibba *et al.* (2020) pointed out that Bayesian multi-trait multi-environment (BMTME; Montesinos-López *et al.* 2016, 2019b,c,d) analysis of multi-trait multi-environment data was useful to select wheat lines for quality traits. These used data from the International Maize and Wheat Improvement Center (CIMMYT) spring wheat breeding program in which 1400 preliminary yield trial (YT) entries are characterized for several quality traits and ∼600 lines are advanced to the next cycle based on yield and quality traits. The study included only wheat quality traits measured in the second stage of testing, with first stage testing done based on single-trait grain yield.

Despite the promise of multi-trait GP, there is a need to evaluate different CV methods to ensure models and methods are accurately and efficiently compared (Runcie and Cheng 2019). In this study, the main objective was to compare the estimates of GP accuracy based on four CV methods, the first two based on the standard Pearson correlation, and the second two based on the Pearson correlations corrected as described by Runcie and Cheng (2019). This comparison used data from Ibba *et al.* (2020), with the addition of another prediction year (2019–2020). The original 13 quality traits measured in each of six pairs of years, plus grain yield, were used. The addition of grain yield to the 13 quality traits is important, as it is the trait measured in the preceding stage of selection and is therefore expected to improve the accuracy of prediction.

## Materials and methods

### Plant material

Spring wheat lines selected for quality and grain yield analyses from CIMMYT first year yield trials (YT) were used as the training population to predict the quality of lines selected from elite yield trials (EYT) for quality and grain yield analyses in a second year. The analyses were conducted for 14 traits defined in Table 1, unless specified differently, and using six sets of data, as reported below:

**Table 1:** Traits evaluated in the six data sets

Number	Trait abbreviation	Name trait	Type of trait
1	ALVPL	Curve configuration ratio, indicative of the ratio between dough tenacity and extensibility	Quality trait
2	ALVW	Dough deformation energy, indicative of the overall gluten strength	Quality trait
3	FLRPRO	Flour protein reported at 14% moisture content	Quality trait
4	FLRSDS	Sodium dodecyl sulfate sedimentation	Quality trait
5	GRNHRD	Grain hardness	Quality trait
6	GRNPRO		Quality trait
7	GY	Grain yield in tons per hectare	Grain trait
8	LOFVOL	Bread loaf volume measured by rapeseed displacement in accordance with AACC method 10-05.01 (AACC, 2010)	Quality trait
9	MIXTIM	Time to peak mixing strength	Quality trait
10	MIXTORQ	Height at the midline of peak mixing strength	Quality trait
11	TESTWT	Test weight in kg hL^−^ was measured using a 37.81-mL sample	Quality trait
12	TKW	1000-kernel weight in grams	Quality trait
13	L	Average abscissa, of rupture, indicative of dough extensibility	Quality trait
14	P	Maximum overpressure, indicative of dough tenacity	Quality trait

- *Data* *1*(2013–2014/2014–2015), 1,301 lines from the 2013–2014 YT and 472 lines from the 2014–2015 EYT trial. In this data set, traits L (average abscissa) and P (maximum overpressure) were not measured, meaning that 11 quality traits and 1 grain yield trait were used.
- *Data* *2* (2014–2015/2015–2016), 1,337 lines from the 2014–2015 YT and 596 lines from the 2015–2016 EYT trial.
- *Data* *3* (2015–2016/2016–2017), 1,161 lines from the 2015–2016 YT and 556 lines from the 2016–2017 EYT trial.
- *Data* *4* (2016–2017/2017–2018), 1,372 lines from the 2016–2017 YT and 567 lines from the 2017–2018 EYT trial.
- *Data* *5* (2017–2018/2018–2019), 1,386 lines from the 2017–2018 YT and 509 lines from the 2018–2019 EYT trial.
- **Data** **6** (2018–2019/2019–2020), 1,276 lines from the 2018–2019 YT and 124 lines from the 2019–2020 EYT trial.

Data sets 1–5 are similar to those used by Ibba *et al.* (2020) but with the addition of grain yield data. Data set 6 is new quality and yield data. All quality analyses were performed according to the methods approved by the AACCI International, or other modified methods described in Battenfield *et al.* (2016). The full names, descriptions and abbreviations of the traits evaluated in the six data sets are provided in Table 1. Further details of how each trait was measured can be found in Ibba *et al.* (2020).

### Genotypic data

All the lines were genotyped using genotyping-by-sequencing (GBS; Poland *et al.* 2012). The TASSEL v.5 (Trait Analysis by Association Evolution and Linkage) GBS pipeline was used to call marker polymorphisms (Glaubitz *et al.* 2014), and a minor allele frequency of 0.01 was used for single nucleotide polymorphism (SNP) discovery. The resulting 6,075,743 unique tags were aligned to the wheat genome reference sequence (RefSeq v.1.0) (IWGSC 2018) with an alignment rate of 63.98%. After filtering for SNPs with homozygosity >80%, *P*-value for Fisher’s exact test <0.001 and χ^2^ value lower than the critical value of 9.2, we obtained 78,606 GBS markers that passed at least one of those filters. These markers were further filtered for less than 50% missing data, greater than a 0.05 minor allele frequency and less than 5% heterozygosity in all the datasets. Markers with missing data were imputed using the “expectation-maximization” algorithm in the “R” package rrBLUP (Endelman 2011).

### Genome-based statistical models

If in each environment i=1,…,I, for each line $j=1,\ldots ,J,\;n_{T}$ traits are measured, $Y_{\mathrm{ijt}}$, $t=1,\ldots ,n_{T}$, a multi-trait genomic linear mixed model is given by

$$Y=1\mu^{T}+X_{E}\beta_{E}+Z_{L}b_{1}+Z_{LE}b_{2}+e\;(1)$$

where $Y={\left[Y_{1}^{T},.,Y_{I}^{T}\right]}^{T}$ is the matrix response values of all traits and all lines in all environments, $Y_{i}={\left[Y_{i1},\ldots ,Y_{iJ}\right]}^{T}$, $Y_{ij}^{T}=\left[Y_{ij1},\ldots ,Y_{ijn_{T}}\right]$, $\mu ={\left(\mu_{1},\ldots ,\mu_{n_{T}}\right)}^{T}$ is the vector with general means for the $n_{T}$ traits, $X_{E}$, is the matrix design of fixed environment effects ($\beta_{E}$), $Z_{L}$ and $Z_{EL}$ are the incident matrix design of random lines ($b_{1}$) and interaction-genotype by environment effects ($b_{2}$), respectively, and e is the error term matrix with a matrix normal distribution $MN\left(0,I_{IJ},R\right)$ and is assumed independently of $b_{1}$ and $b_{2}$, which have distributions $MN_{J\times n_{T}}\left(0,G,\Sigma_{T}\right)\;$and $MN_{IJ\times n_{T}}\left(0,I_{I}⊗G,\Sigma_{T}\right)$, where $I_{I}$ is the identity matrix of dimension I×I, R and $\Sigma_{T}$ are the positively defined matrices of dimension $n_{T}\times n_{T},\;⊗$ the Kronecker product and G is the genomic relationship matrix of dimension J×J and was computed as suggested by VanRaden (2008).

A Bayesian estimation of this model can be achieved by assuming the following priors: $f\left(\mu ,\;\mathrm{vec}\left(\beta_{E}\right)\;\right)\propto 1$, and independent distributions for the covariance matrices of residuals R and for $\Sigma_{T}$, $\Sigma_{T}∼IW\left(v_{T},S_{T}\right)$ and $R∼IW\left(v_{R},S_{R}\right)$, where $\mathrm{vec}\left(\cdot \right)$ and IW denote the vectorization operation and the inverse Wishart distribution. This model was implemented with the Multitrait function in the BGLR R package version GitHub: https://github.com/gdlc/BGLR-R (Pérez and de los Campos 2014) that is considered work in progress where the hyper-parameters for the priors could be modified in the future.

### Derivation of the corrected Pearson’s correlation

$\mathrm{If}\;Y_{\mathrm{tst}}=\mu +g_{\mathrm{tst}}+\epsilon_{\mathrm{tst}}$ is the phenotypic response of a line in testing data, then ${\hat{g}}_{\mathrm{tst}}$ is a prediction of the genotypic effect of this same line obtained with only the information of training data. Therefore, because $\mathrm{Cov}\left({\hat{g}}_{\mathrm{tst}},\epsilon_{\mathrm{tst}}\right)=0,$ the correlation of the ${\hat{g}}_{tst}$ with the phenotypic response can be expressed as

$$\mathrm{Cor}\left({\hat{g}}_{\mathrm{tst}},Y_{\mathrm{tst}}\right)=\frac{\mathrm{Cov}\left({\hat{g}}_{\mathrm{tst}},Y_{\mathrm{tst}}\right)}{\sqrt{\mathrm{Var}\left({\hat{g}}_{\mathrm{tst}}\right)Var\left(Y_{\mathrm{tst}}\right)}}=\frac{\mathrm{Cov}\left({\hat{g}}_{\mathrm{tst}},\mu +g_{\mathrm{tst}}+\epsilon_{\mathrm{tst}}\right)}{\sqrt{\mathrm{Var}\left({\hat{g}}_{\mathrm{tst}}\right)Var\left(Y_{\mathrm{tst}}\right)}}=\frac{\mathrm{Cov}\left({\hat{g}}_{\mathrm{tst}},g_{\mathrm{tst}}\right)+\mathrm{Cov}\left({\hat{g}}_{\mathrm{tst}},\epsilon_{\mathrm{tst}}\right)}{\sqrt{\mathrm{Var}\left({\hat{g}}_{\mathrm{tst}}\right)Var\left(Y_{\mathrm{tst}}\right)}}=\frac{\mathrm{Cov}\left({\hat{g}}_{\mathrm{tst}},g_{\mathrm{tst}}\right)}{\sqrt{\mathrm{Var}\left({\hat{g}}_{\mathrm{tst}}\right)Var\left(Y_{\mathrm{tst}}\right)}}=\frac{\mathrm{Cov}\left({\hat{g}}_{\mathrm{tst}},g_{\mathrm{tst}}\right)}{\sqrt{\mathrm{Var}\left({\hat{g}}_{\mathrm{tst}}\right)Var\left(g_{\mathrm{tst}}\right)}}\sqrt{\frac{\mathrm{Var}\left(g_{\mathrm{tst}}\right)}{\mathrm{Var}\left(Y_{\mathrm{tst}}\right)}}=\mathrm{Cor}\left({\hat{g}}_{\mathrm{tst}},g_{\mathrm{tst}}\right)\sqrt{h^{2}}$$

where $h^{2}=\frac{\mathrm{Var}\left(g_{\mathrm{tst}}\right)}{\mathrm{Var}\left(Y_{\mathrm{tst}}\right)}$ is the heritability.

Motived by the derivation above and from the results that stated that the sample correlation between the phenotypic values and the estimated breeding values of lines to be predicted (testing data) divided by the square-root of heritability, $\mathrm{Cor}\left(\hat{g},y\right)/\sqrt{h^{2}}$, is an approximate unbiased estimator for the correlation between true and predicted breeding value$\;\mathrm{Cor}({\hat{g}}_{\mathrm{tst}},g_{\mathrm{tst}})\;$(Daetwyler *et al.* 2013; Runcie and Cheng 2019), $h^{2}$ was computed using the whole data set, next we described the way to calculate the different corrected Pearson’s correlation between observed and predicted values. We study the Runcie and Cheng (2019) which is referred as *PC*3 or method 3.

### Calculating corrected Pearson correlations (PC)

#### PC1 (method 1) and PC2 (method 2)

The standard correlation between observed phenotypic values and the predicted breeding values will be referred as PC1 (method 1, or predictive ability). As described above, this standard correlation between observed phenotypic values and the predicted breeding values divided by square-root of heritability, will be referred as PC2 (method 2, or predictive accuracy). Note that *PC*2 is a nonparametric estimator of the genetic correlation (correlation between the unknown true genetic value with the estimate genetic value).

#### PC3 (method 3)

The calculation of $PC_{3}$ is based on fitting the following bivariate genomic model:

YioYip=μoμp+giogip+eioeip

where $Y_{io}$ and $Y_{ip}$, $i=1,\ldots ,\;n_{\mathrm{tst}}$ are the observed phenotype values and their corresponding predicted values (under a multi-trait or under a single trait model) of a trait of interest, respectively, in the testing data, $g={\left(g_{1},\ldots ,g_{n_{\mathrm{tst}}}\right)}^{T}∼N\left(0_{n_{\mathrm{tst}}},G\;\Sigma \right)$, $\Sigma =\left[\begin{array}{cc} \sigma_{go}^{2} & \sigma_{\mathrm{gop}}^{2}\\ \sigma_{\mathrm{gpo}}^{2} & \sigma_{gp}^{2} \end{array}\right]$, $e_{i}={\left(e_{io},e_{ip}\right)}^{T}∼N_{2}\left(0_{2},R\right)$ and $R=\left[\begin{array}{cc} \sigma_{eo}^{2} & \sigma_{\mathrm{eop}}^{2}\\ \sigma_{\mathrm{epo}}^{2} & \sigma_{ep}^{2} \end{array}\right]$. Having estimated the parameter of this model, the $PC_{3}$ is calculated as:

$$PC_{3}=\frac{{\hat{\sigma }}_{\mathrm{gop}}^{2}}{{\hat{\sigma }}_{go}^{2}+{\hat{\sigma }}_{gp}^{2}}\sqrt{{\hat{h}}_{p}^{2}}$$

where ${\hat{h}}_{p}^{2}=\frac{{\hat{\sigma }}_{gp}^{2}}{{\hat{\sigma }}_{gp}^{2}+{\hat{\sigma }}_{ep}^{2}}$ is the estimated heritability of the predicted trait value ($Y_{p}$) under this auxiliar bivariate model. The $PC_{3}$ is the corrected Pearson’s correlation referred as method 3 and is considered the parametric estimate the correlation between the true and predicted breeding values $\left[\mathrm{Cor}(g_{\mathrm{tst}},\;{\hat{g}}_{\mathrm{tst}},\right)$].

To obtain the value of $PC_{3}$ the package MCMCglmm (Hadfield, 2010) will be used by setting the prior for R as list(V = diag(c(.5,.01),2),nu = 3), and the prior for Σ as list(V = diag(c(.5,.5),2), nu = 3, alpha.mu = rep(0,2), alpha. V = diag(1,2)). This gave an approximately uniform distribution on the genetic correlation. For the residual covariance, we specified $\sigma_{\mathrm{eop}}^{2}=0$ by specifying rcov = ∼idh(trait):units because there is no nongenetic correlation between $Y_{io}$ and $Y_{ip}$ in this experiment. The prior mean for the $\sigma_{\mathrm{epp}}^{2}$ was set close to 0 because this parameter is expected to be very small when the predictions $Y_{ip}$ are posterior means of a parameter in the original model with covariance **G**.

#### PC4 (method 4)

Because, $\mathrm{Cor}\left({\hat{g}}_{\mathrm{tst}},Y_{\mathrm{tst}}\right)=\mathrm{Cor}\left({\hat{g}}_{\mathrm{tst}},g_{\mathrm{tst}}\right)\sqrt{h^{2}}$, then if an estimate for $\mathrm{Cor}\left({\hat{g}}_{\mathrm{tst}},g_{\mathrm{tst}}\right)$ is given, then by multiplying this by $\sqrt{h^{2}}$, an estimate for $\mathrm{Cor}\left({\hat{g}}_{\mathrm{tst}},Y_{\mathrm{tst}}\right)$ is obtained. This uncorrected Pearson’s correlation ($PC_{4}$) will be denoted as method 4.

In order to evaluate the prediction performance, we compared PC1 to PC4 along with the mean squared error of prediction (MSEP) computed between the observed and predicted testing values in each partition.

### Cross-validation strategy

Since each data set contains information for two breeding cycles (previous and current), the evaluation of the prediction performance of model (1) was carried out using a cross-validation strategy that consisted of predicting 90% (testing; current cycle) of lines with the full information of the previous cycle, plus the remaining 10% of the current cycle, which allows us to estimate the environmental effects included in the model. The selection of 10% of current lines for inclusion in the training set was random and represents a proportion of material which could be rapidly tested prior to post-harvest selection decisions being finalized. This random selection was performed with five-fold cross-validation, resulting in five different values of each metric used, and from which the average was reported as the prediction performance.

## Results

Several phenotypic correlations were recorded between the traits measured for each data set. Table 2 displays the phenotypic correlations between the traits under study between data sets 1 and 2 with the remaining correlations between datasets given in Appendix Table A1 (Phenotypic Pearson’s correlation of data sets 3 and 4) and Table A2 (Phenotypic Pearson’s correlation of data sets 4 and 5).

**Table 2:** Raw phenotypic sample correlation matrix between traits based on all information of data set 1 (values in upper triangular table) and data set 2 (values in lower triangular table)

	TESTWT	TKW	GRNHRD	GRNPRO	FLRPRO	FLRSDS	MIXTIM	MIXTORQ	ALVW	ALVPL	LOFVOL	L	P	GY
TESTWT	1.00	0.25	−0.32	0.01	0.02	0.00	−0.17	−0.15	−0.05	0.14	0.06	—	—	0.35
TKW	0.36	1.00	−0.27	−0.06	−0.04	0.00	−0.22	−0.21	−0.12	0.14	−0.06	—	—	0.25
GRNHRD	−0.60	−0.43	1.00	0.01	−0.03	0.09	0.11	0.10	−0.01	−0.33	0.06	—	—	−0.34
GRNPRO	−0.02	0.03	−0.10	1.00	0.90	0.34	−0.01	0.05	0.21	−0.23	0.55	—	—	−0.17
FLRPRO	−0.14	−0.06	0.00	0.92	1.00	0.39	−0.01	0.05	0.24	−0.20	0.59	—	—	−0.15
FLRSDS	0.14	0.09	−0.24	0.43	0.40	1.00	0.36	0.40	0.56	−0.06	0.52	—	—	0.03
MIXTIM	−0.10	−0.19	0.01	−0.05	−0.02	0.38	1.00	0.98	0.84	0.19	0.18	—	—	−0.28
MIXTORQ	−0.02	−0.12	−0.08	0.03	0.05	0.48	0.97	1.00	0.88	0.20	0.23	—	—	−0.31
ALVW	0.13	0.02	−0.26	0.23	0.21	0.64	0.80	0.88	1.00	0.26	0.38	—	—	−0.17
ALVPL	0.45	0.35	−0.56	−0.13	−0.21	0.12	0.16	0.26	0.39	1.00	−0.34	—	—	0.23
LOFVOL	−0.30	−0.26	0.20	0.49	0.54	0.51	0.34	0.36	0.39	−0.38	1.00	—	—	−0.02
L	−0.36	−0.30	0.39	0.31	0.38	0.28	0.20	0.16	0.13	−0.75	0.65	—	—	—
P	0.44	0.30	−0.57	0.01	−0.07	0.40	0.43	0.54	0.73	0.88	−0.11	−0.49	1.00	—
GY	0.54	0.33	−0.51	−0.14	−0.28	0.08	−0.10	−0.07	0.07	0.44	−0.31	−0.37	0.40	1.00

The corresponding values for traits L and P in data set 1 are missing.

### Differences between predictive performance of four methods

**Figure 1:**
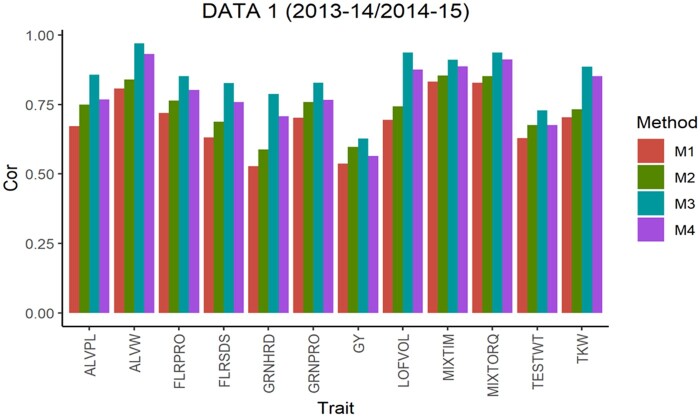
Data 1 (2013–2014/2014–2015). Average Pearson’s correlation (Cor) computed with four methods. Method 1 (M1): Pearson’s correlation between observed phenotypic values and predicted breeding values. Method 2 (M2): Pearson’s correlation computed dividing method 1 by the square-root of the heritability. Method 3 (M3): Corrected Pearson’s correlation proposed by Runcie and Cheng (2019). Method 4 (M4): Pearson’s correlation computed multiplying method 3 by $\sqrt{h^{2}}.[\mathrm{AQ}9]$

**Figure 2:**
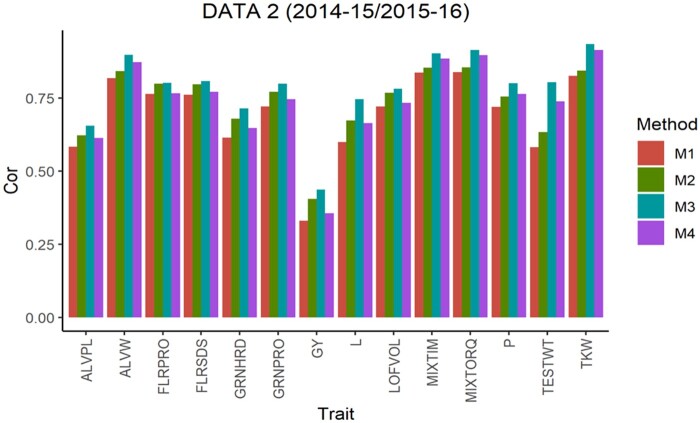
Data 2 (2014–2015/2015–2016). Average Pearson’s correlation (Cor) computed with four methods. Method 1 (M1): Pearson’s correlation between observed phenotypic values and predicted breeding values. Method 2 (M2): Pearson’s correlation computed dividing method 1 by the square-root of the heritability. Method 3 (M3): Corrected Pearson’s correlation proposed by Runcie and Cheng (2019). Method 4 (M4): Pearson’s correlation computed multiplying method 3 by $\sqrt{h^{2}}.$

**Figure 3:**
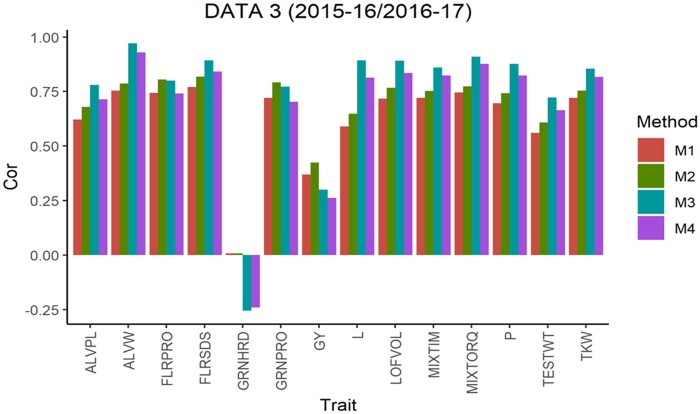
Data 3 (2015–2016/2016–2017). Average Pearson’s correlation (Cor) computed with four methods. Method 1 (M1): Pearson’s correlation between observed phenotypic values and predicted breeding values. Method 2 (M2): Pearson’s correlation computed dividing method 1 by the square-root of the heritability. Method 3 (M3): Corrected Pearson’s correlation proposed by Runcie and Cheng (2019). Method 4 (M4): Pearson’s correlation computed multiplying method 3 by $\sqrt{h^{2}}.$

**Figure 4:**
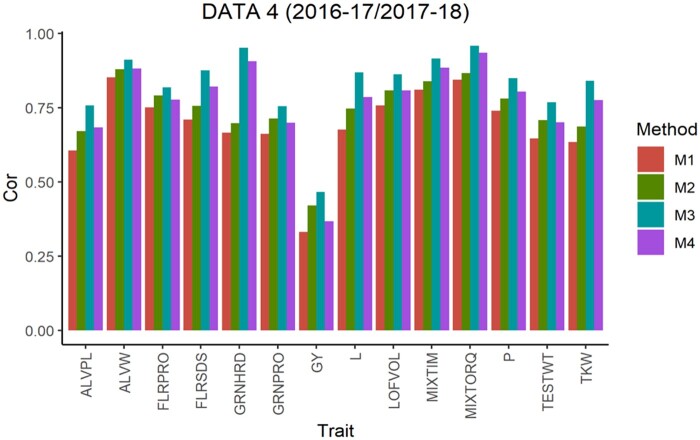
Data 4 (2016–2017/2017–2018). Average Pearson’s correlation (Cor) computed with four methods. Method 1 (M1): Pearson’s correlation between observed phenotypic values and predicted breeding values. Method 2 (M2): Pearson’s correlation computed dividing method 1 by the square-root of the heritability. Method 3 (M3): Corrected Pearson’s correlation proposed by Runcie and Cheng (2019). Method 4 (M4): Pearson’s correlation computed multiplying method 3 by $\sqrt{h^{2}}.$

**Figure 5:**
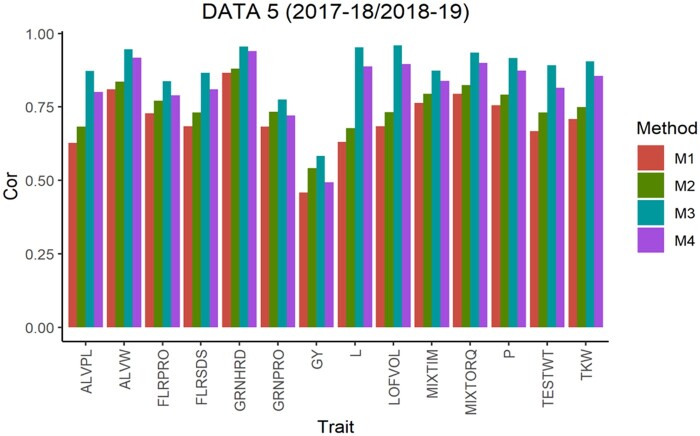
Data 5 (2017–2018/2018–2019). Average Pearson’s correlation (Cor) computed with four methods. Method 1 (M1): Pearson’s correlation between observed phenotypic values and predicted breeding values. Method 2 (M2): Pearson’s correlation computed dividing method 1 by the square-root of the heritability. Method 3 (M3): Corrected Pearson’s correlation proposed by Runcie and Cheng (2019). Method 4 (M4): Pearson’s correlation computed multiplying method 3 by $\sqrt{h^{2}}.$

**Figure 6:**
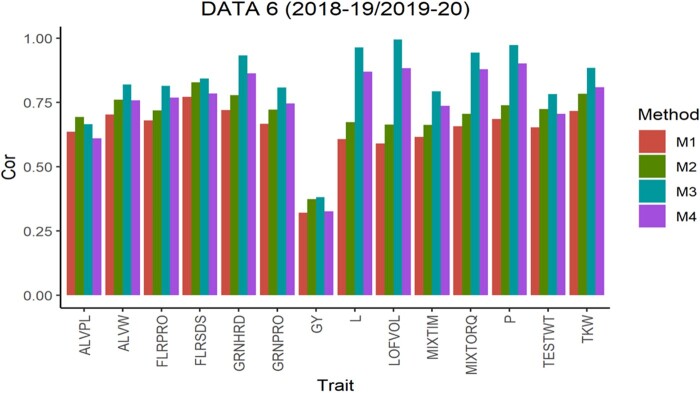
Data 6 (2018–2019/2019–2020). Average Pearson’s correlation (Cor) computed with four methods. Method 1 (M1): Pearson’s correlation between observed phenotypic values and predicted breeding values. Method 2 (M2): Pearson’s correlation computed dividing method 1 by the square-root of the heritability. Method 3 (M3): Corrected Pearson’s correlation proposed by Runcie and Cheng (2019). Method 4 (M4): Pearson’s correlation computed multiplying method 3 by $\sqrt{h^{2}}.$

The average Pearson’s correlation (APC) for all methods across the 5-testing set configurations are shown in Figures 1–6, for the 6 pairs of years. From this, we observe that in data sets 1, 2, 4 and 5 for all traits method 3 shows the largest values as well as for 10 of the 14 traits in data set 3 and 13 of 14 traits in data set 6. For the traits where method 3 was not superior, the APC corresponding to method 2 showed the highest values with a similar result observed across data sets. For traits ALVW, FLRSDS, L, LOFVOL, MIXTIM, MIXTORQ, P, TESTWT, and TKW in all data sets, method 3 gave the highest values of APC. For traits ALVPL, FLRPRO, GRNHRD, GRNPRO, and GY in 5 out 6 data sets method 3 also gave the best performance. Where these traits were missing, method 2 resulted in better performance.

**Figure 7:**
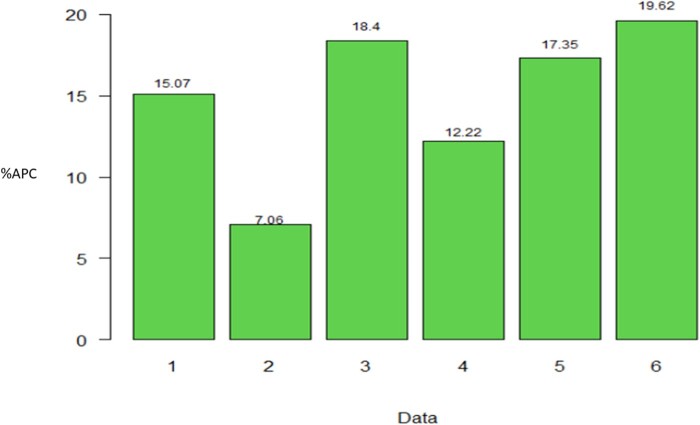
Percentage of relative differences between the average Pearson’s correlation (%APC) of method 3 with regard to the APC of method 2, across traits where the former resulted in larger APC values, for each data set (data 1–6). For the data set 1, 2, 4, and 5 in all traits the APC value of method 3 was larger than APC of method 2, and also this happened in 10 and 13 out 14 traits, for data sets 3 and 6, respectively.

Taken across all traits, method 3 gave the highest APC. In each data set, the average APC difference of method 3 compared to method 2 was 15.07 (data set 1), 7.06 (data set 2), 18.04 (data set 3), 12.22 (data set 4), 17.35 (data set 5) and 19.62% (data set 6); in data set 3 the corresponding APC value for trait GRNHRD was not considered. Therefore, considering traits where method 3 gave the best performance, the difference between method 3 and the commonly used Pearson’s correction method (method 2) across all traits ranged from 7.06% to 19.62% (Figure 7).

**Figure 8:**
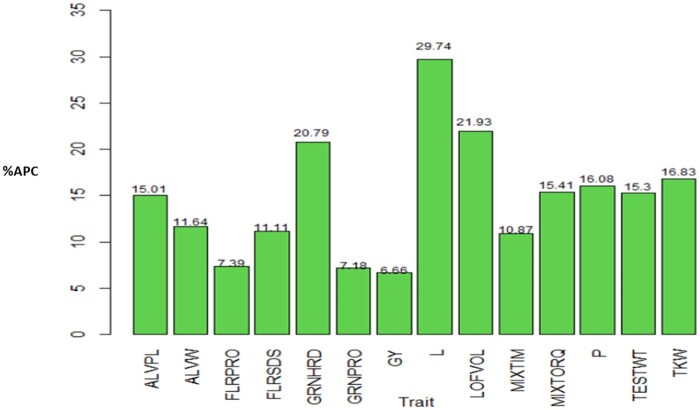
Percentage of relative differences between the average Pearson’s correlation (%APC) of method 3 with regard to the APC of method 2 across data sets where APC of the former method (M3) was larger than APC of method 2, for each trait. For traits ALVPW, FLRSDS, L, LOFVOL, P, MIXTIM, MIXTORQ, TESTWT, and TKW in all data sets method 3 show a larger APC value compared with method 2, and for traits ALVPL, FLRPRO, GRNHRD, GRNPRO, and GY, in 5 out data sets the method 3 was superior: in data sets 1, 2, 4, 5, and 6 for the last three of these traits and in the first five data set for trait ALVPL.

On a trait basis across all data sets, method 3 estimated a higher prediction accuracy (in terms of APC) compared to method 2. The average APC of method 3 was larger than the corresponding APC value of method 2 by 15.01, 11.64, 7.39, 11.11, 20.79, 7.18, 6.66, 29.74. 21.93, 10.87, 15.41, 16.08, 15.3, and 16.83% for traits ALVPL, ALVW, FLRPRO, FLRSDS, GRNHRD, GRNPRO, GY, L, LOFVOL, MIXTIM, MIXTORQ, P, TESTWT, TKW, respectively (Figure 8). These results mean that, in general across data sets, the APC for method 3 is higher than the APC of method 2 by 6.66% to 29.74%. In the cases where the APC of method 3 was observed to be better than APC of method 2, the traits in which the smaller and larger increase happened were different for each data set: for data set 1, it was GY and GRNHRD; for data set 1 it was in traits FLRPRO and TESTWT; for data set 3 FLRSDS and L; for data set 4, FLRPRO and GRNHRD; for data set 5, GRNPRO and L; and for data set 6, FLRSDS and LOFVOL.

As previously described, Figures 1–6 also report the APC values obtained with the “uncorrected” version of the Pearson’s correlation (methods 1 and 4). These were also used to estimate the correlation between the predicted and true breeding values. These are the corrected versions obtained with methods 2 and 3, respectively, but multiplied by the square root of the heritability, and therefore no further comparisons are made between the uncorrected versions of methods 1 and 4.

**Figure 9:**
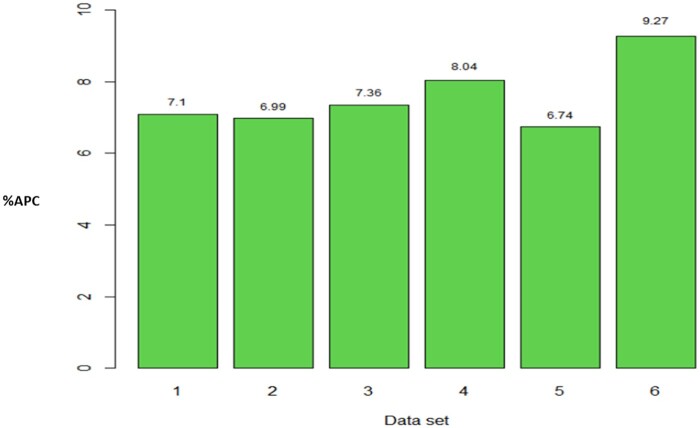
Average across traits of the percentage of relative difference of average Pearson’s correlation (%APC) of method 2 (or method 3) with regard to the average Pearson’s correlation (APC) of method 1 (or method 4):$\frac{1}{\sqrt{h^{2}}}-1$ for data 1–6.

**Figure 10:**
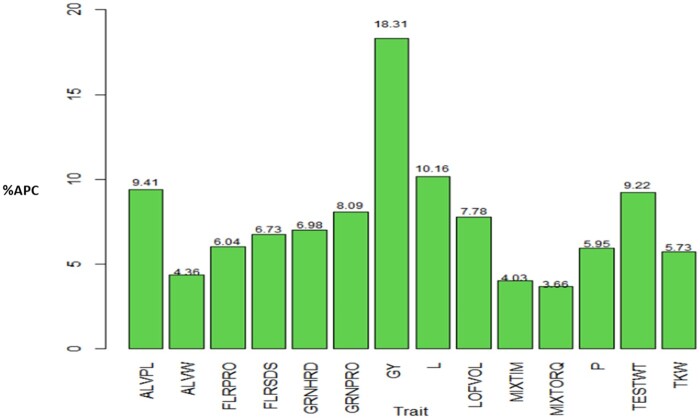
Average across traits of the percentage of relative difference of average Pearson’s correlation (%APC) obtained with method 2 (or method 3) with regard to the APC obtained with method 1 (or method 4): $1/\sqrt{h^{2}}-1.$

However, it is interesting to compare the relative differences in terms of prediction performance for APC under the corrected and not corrected versions (*e.g.*, method 1 *vs* method 2; method 3 *vs* method 4). Because the APC of methods 1 and 4 can be obtained by multiplying by the square root of the heritability by the APC of methods 2 and 3, then the APC of methods 1 and 4 will not be superior to the APC of methods 2 and 3, respectively, and in general, the relative difference is equal to $1/\sqrt{h^{2}}-1$. In Figure 9, across traits we observe the difference in predictions for each data set of method 3 (method 2) with regard to the method 4 (method 1), where the smallest difference was observed to be 6.74% (data set 5) and the largest was 9.27% (data set 6). In turn, Figure 10 shows, for each trait across environments, the relative differences between method 3 (method 2) and method 4 (method 1), where we observe that the smallest difference was 3.66% (trait MIXTORQ), whilst the largest was of 18.31% for GY.

### Multi-trait genomic models outperform single-trait models

In order to compare predictive performance, the multi-trait model (1) was trained using the 14 traits in all six data sets (except for data set 1 which only contained 12 traits). In the testing set, it was assumed that all traits were missing and should be predicted. In the single-trait model, we trained the model with grain yield and the predictive performance of the test set was compared with the predictive performance for yield from the multi-trait model. In both multi-trait and single-trait models, the same predictors were used. Table 3 shows the resulting prediction performance with both models, including the APC under the four methods described previously and the average mean square error of prediction (MSEP) for both models.

**Table 3:** Average Pearson’s correlation (APC) for the four methods, method 1 (M1), method 2 (M2), method 3 (M3), and method 4 (M4), calculated as metrics for prediction performance for each data set, all metrics calculated under the model (1) (multi-trait) and under a univariate genomic model with the same predictors as model given in equation (1) and GY as response (single-trait)

	Multi-trait					Single-trait				
	M1	M2	M3	M4		M1	M2	M3	M4	
Data	APC (SD)	APC (SD)	APC (SD)	APC (SD)	MSEP (SD)	APC (SD)	APC (SD)	APC (SD)	APC (SD)	MSEP (SD)
1	0.5376 (0.0177)	0.5973 (0.0197)	0.628 (0.0473)	0.5652 (0.0426)	0.1354 (0.0056)	0.5600 (0.0144)	0.5908 (0.0152)	0.6395 (0.0361)	0.6062 (0.0343)	0.1313 (0.0026)
2	0.3307 (0.0212)	0.4053 (0.026)	0.4363 (0.017)	0.356 (0.0139)	0.1335 (0.004)	0.2623 (0.0343)	0.2865 (0.0375)	0.3425 (0.0634)	0.3136 (0.058)	0.1391 (0.0061)
3	0.3691 (0.0305)	0.4236 (0.035)	0.2999 (0.0263)	0.2613 (0.0229)	0.2303 (0.0037)	0.3421 (0.0563)	0.3639 (0.0599)	0.2333 (0.0502)	0.2193 (0.0472)	0.2352 (0.0066)
4	0.3315 (0.0228)	0.4202 (0.0289)	0.4663 (0.0362)	0.3679 (0.0286)	0.1581 (0.0116)	0.283 (0.0282)	0.3097 (0.0308)	0.3822 (0.0918)	0.3492 (0.0839)	0.1639 (0.0172)
5	0.4589 (0.0352)	0.5414 (0.0415)	0.5827 (0.0513)	0.4939 (0.0434)	0.2065 (0.0158)	0.398 (0.0479)	0.4253 (0.0512)	0.4699 (0.0351)	0.4398 (0.0328)	0.2165 (0.02)
6	0.3209 (0.0289)	0.3743 (0.0337)	0.3814 (0.0499)	0.327 (0.0428)	0.1246 (0.0079)	0.3094 (0.0436)	0.3425 (0.0482)	0.3429 (0.0757)	0.3097 (0.0684)	0.1237 (0.0089)

MSEP is the average mean square error of prediction. Its standard deviation across the five partition is given is in parenthesis (SD).

This shows that across data sets and methods, the multi-trait model outperformed the single-trait model in the prediction of grain yield. The only exception is the first data set, where the single-trait model was better with methods 1, 3, and 4. The multi-trait model also gave better MSEP than the single-trait model in 4 of the data sets, while in the first and last data sets the single-trait model was better. For APC under method 1 the smallest and largest gains of the multi-trait model were observed in data sets 6 and 2, with 3.70% and 26.09%, respectively. Under method 2, the smallest and largest gains of the multivariate were 1.08% (data set 1) and 41.51% (data set 2), respectively. With method 3, 11.24 (data set 6) and 28.54% (data set 3), and with method 4, the smallest gain was obtained in data 4 (5.34%), while the largest gain in data set 3 (19.14%).

Likewise, Table 3 shows that the multivariate model gives better performance in terms of MSEP for all data sets except in data set 1 and 6; the average of this difference across the data sets where the multi-trait model was superior is 3.73%. For MSEP, the lowest and highest improvements were observed in data set 3 (2.12%) and 5 (4.88%). Furthermore, the relative difference of MSEP with the single-trait model with regard to the multivariate model in data set 1 was of 3.11%, and in data set 6 this difference was 0.70%. Across all metrics, the prediction of GY improved moderately when using the multi-trait genomic model when compared to the single-trait model.

Finally, we compared the relative difference in terms of prediction performance between method 1 and method 2 or between method 3 and method 4, for which the relative difference is $\frac{1}{\sqrt{h^{2}}}-1$. For the multi-trait results, method 2 (method 3) was superior to method 1 (method 4) by 11.10, 22.56, 14.79, 26.72, 17.98, and 16.67% for data sets 1, 2, 3, 4, 5, and 6, respectively, and across data sets at an average of 18.31%. Under the single-trait model, method 2 (method 3) was superior to method 1 (method 4) by 5.5, 9.22, 6.37, 9.47, 6.86, and 10.73% for data sets 1, 2, 3, 4, 5, 6, respectively, and on average 8.02% across data sets.

## Discussion

Despite the benefit of performing multi-trait analyses, multi-trait models are computationally intensive and complex. Varying trait response patterns also create very complex genotype × environment interactions (G × E). Multi-trait models could also increase convergence problems when fitted with classical methods (like maximum likelihood or restricted maximum likelihood; Montesinos-López *et al.* 2019c).

Two main factors affecting the efficiency of GP are the phenotype heritabilities and the choice of the training population in relation to the test population (Crossa *et al.* 2017). Wheat quality traits mostly exhibit moderate to high narrow-sense heritability (*h*^2^) values (Ibba *et al.* 2020). Battenfield *et al.* (2016) showed that in wheat, a higher prediction accuracy was obtained when increasing the size of the training population over years from 250 to 4095 wheat lines. However, obtaining a large training population for multi-trait wheat quality traits is expensive, therefore, the wheat multi-trait quality information produced each breeding cycle can be used to develop GP models that may help reduce the number of lines for quality trait analyses. This would reduce the cost of wheat quality analyses by discarding lines predicted to have undesirable quality traits and keep only the lines that are promising for their processing traits. For example, Ibba *et al.* (2020) evaluated the prediction performance of two multi-trait models for 13 wheat quality traits using five data sets from lines evaluated in the field during two consecutive years. Lines in the second year (testing) were predicted using the quality information obtained in the first year (training) and showed moderate to high prediction accuracies for most of the quality traits.

Since genomic selection is a predictive methodology, it is therefore crucial to adequately select: the model (Bayesian or nonBayesian, linear or nonlinear, single- or multi-trait, and so on.), the training set, and the traits of interest. Each of these elements contributes in different ways to successful implementation of GS and is assessed subsequently in terms of metrics to evaluate predictive performance (MSEP, Pearson’s correlation, MAAPE).

The two most popular metrics for assessing predictive performance are the Pearson’s correlation and the correlation divided by the square root of the heritability. However, empirical research shows that the computation of both metrics underestimates the true correlation between the estimated breeding values and the observed phenotype. For this reason, using real data sets, we illustrated the calculation of the corrected Pearson’s correlation, and compared it with the conventional Pearson’s correlation (Daetwyler *et al.* 2008).

We observed that the conventional Pearson’s correlation underestimates the value of the true correlation and is very conservative regarding the true genetic correlation. When comparing the predictive performance between methods, method 4 underestimated the accuracy because the relative difference between these methods is equal to $1/\sqrt{h^{2}}-1$. Method 2 and 3 are reasonable estimates of the prediction accuracy of genetic values represented by $\mathrm{Cor}(g_{\mathrm{tst}},\;{\hat{g}}_{\mathrm{tst}},)$ and are considerably higher than method 1 which seems to clearly underestimate $\mathrm{Cor}(g_{\mathrm{tst}},\;{\hat{g}}_{\mathrm{tst}})$. Although the mathematical derivation of method 3 is reasonable and current results of data used in this study suggest higher prediction accuracy than method 2, we propose that further investigations by means of computer simulations are required to model different heritability’s and distribution of markers along with phenotypic effects. This will further clarify the role of method 3 for estimating the prediction accuracy of the correlation between the true and estimated genetic value. More empirical, simulation and analytical studies are necessary for deriving the expected values of each method for computing the correlation, the precision of correlation estimates and the degree of unbiasedness of each method.

Taken together our result indicate that the accuracy of GP should be reported with the corrected Pearson’s correlation of method 3, as proposed by Runcie and Cheng (2019). This method calculates the genetic correlation between the predicted breeding value and true breeding value by fitting a bivariate model in which the phenotypic values correspond to one trait and its corresponding predicted breeding values corresponds to the second trait. This corrects the underestimation obtained when the commonly used correction method of the standard Pearson’s is applied (method 2).

We also found that a multi-trait model outperforms a single-trait model in the prediction of grain yield [range of difference between 1.08 and 41.48% (Pearson’s correlation), and between 2.12 and 4.88 in terms of MSEP]. These results agree with previous reports showing that multi-trait models outperform the predictions of single-trait models (Montesinos-López *et al.* 2016, 2018b, 2019a,c; Schulthess *et al.* 2018) when the correlation between traits is moderate or high.

## Conclusions

Overall, our results suggest that the Runcie and Cheng (2019) correction method should be applied to the assessment of predictive performance and that the use of multiple traits from different stages of a breeding program can be incorporated in a multi-trait model to improve predictions. This has implications for the use of multi-trait data for genomic-assisted improvement in wheat breeding programs.

## Author contributions

J.C. provided the initial ideas of the research article together with O.A.M.L. and AML. D.E.R. outlined the main details of method 3 and other theoretical details. O.A.M.L., A.M.L., D.E.R., and J.C. prepared the first draft versions of the article. Several other versions of the article were read, corrected, and completed by L.C., M.I.I., A.R.B., and P.P.R. All authors contributed to the article and approved the submitted version.

## Data availability

Complete phenotypic and genotypic data of the data sets 1–6 each comprising two years are available here: https://hdl.handle.net/11529/10548572.

## Appendix A. Phenotypic Pearson’s correlation of data sets 3–6.

**Table A1:** Raw phenotypic sample correlation matrix between traits based on all information from data set 3 (values in upper triangular table) and data set 4 (values in lower triangular table)

	TESTWT	TKW	GRNHRD	GRNPRO	FLRPRO	FLRSDS	MIXTIM	MIXTORQ	ALVW	ALVPL	LOFVOL	L	P	GY
TESTWT	1.00	0.07	−0.78	−0.23	0.13	0.27	−0.20	−0.20	−0.01	−0.08	0.16	0.11	−0.06	0.64
TKW	0.04	1.00	−0.12	0.02	0.04	−0.06	−0.10	−0.09	−0.06	0.06	−0.11	−0.08	0.02	0.03
GRNHRD	0.05	−0.51	1.00	0.34	−0.09	−0.37	0.19	0.19	−0.04	0.09	−0.21	−0.15	0.05	−0.75
GRNPRO	0.08	0.13	0.02	1.00	0.86	0.25	0.08	0.18	0.30	−0.04	0.42	0.22	0.14	−0.41
FLRPRO	0.12	0.17	−0.08	0.93	1.00	0.48	−0.02	0.09	0.34	−0.09	0.57	0.33	0.13	−0.06
FLRSDS	−0.01	0.02	−0.05	0.35	0.43	1.00	0.28	0.35	0.57	−0.09	0.65	0.47	0.25	0.27
MIXTIM	−0.07	−0.17	0.19	−0.17	−0.23	0.35	1.00	0.96	0.78	0.27	0.23	0.13	0.57	−0.21
MIXTORQ	−0.04	−0.13	0.18	−0.07	−0.10	0.45	0.97	1.00	0.86	0.33	0.28	0.12	0.66	−0.23
ALVW	−0.02	−0.09	0.17	0.16	0.15	0.61	0.82	0.89	1.00	0.38	0.45	0.17	0.78	−0.08
ALVPL	0.09	0.02	0.23	−0.01	0.00	0.05	0.15	0.21	0.27	1.00	−0.33	−0.77	0.85	−0.12
LOFVOL	−0.06	−0.08	−0.06	0.47	0.46	0.58	0.29	0.35	0.48	−0.21	1.00	0.68	0.03	0.08
L	−0.14	−0.05	−0.19	0.16	0.14	0.35	0.25	0.24	0.26	−0.76	0.54	1.00	−0.42	0.09
P	0.06	−0.03	0.24	0.09	0.09	0.43	0.58	0.67	0.78	0.77	0.16	−0.33	1.00	−0.13
GY	−0.03	−0.10	0.11	−0.23	−0.27	−0.04	0.11	0.08	0.01	0.05	−0.09	−0.05	0.03	1.00

**Table A2:** Raw phenotypic sample correlation matrix between traits based on all information from data set 5 (values in upper triangular table) and data set 6 (values in lower triangular table)

	TESTWT	TKW	GRNHRD	GRNPRO	FLRPRO	FLRSDS	MIXTIM	MIXTORQ	ALVW	ALVPL	LOFVOL	L	P	GY
TESTWT	1.00	−0.02	0.07	0.07	0.08	−0.04	−0.02	0.00	−0.01	0.01	−0.01	−0.04	0.01	−0.10
TKW	0.01	1.00	−0.37	0.03	0.04	−0.18	−0.24	−0.22	−0.23	−0.07	−0.21	−0.07	−0.17	0.03
GRNHRD	−0.01	−0.57	1.00	0.04	0.10	0.16	0.27	0.31	0.32	0.36	0.01	−0.20	0.43	−0.07
GRNPRO	0.02	0.06	0.02	1.00	0.84	0.43	−0.13	−0.01	0.26	−0.11	0.42	0.28	0.09	−0.09
FLRPRO	0.08	0.09	0.00	0.92	1.00	0.49	−0.09	0.01	0.27	−0.12	0.49	0.32	0.08	−0.15
FLRSDS	−0.08	−0.20	0.19	0.47	0.50	1.00	0.42	0.50	0.68	0.06	0.63	0.39	0.43	0.02
MIXTIM	−0.04	−0.21	0.17	−0.08	−0.06	0.45	1.00	0.97	0.79	0.31	0.32	0.13	0.64	0.05
MIXTORQ	−0.05	−0.19	0.19	0.04	0.06	0.53	0.97	1.00	0.86	0.37	0.35	0.11	0.73	0.04
ALVW	−0.05	−0.20	0.21	0.25	0.30	0.68	0.83	0.89	1.00	0.36	0.49	0.21	0.81	0.01
ALVPL	0.01	−0.11	0.32	—	−0.03	0.13	0.27	0.32	0.34	1.00	−0.25	−0.75	0.82	0.05
LOFVOL	−0.05	−0.20	0.03	0.33	0.39	0.67	0.31	0.34	0.48	−0.13	1.00	0.61	0.14	−0.09
L	−0.06	−0.02	−0.22	0.20	0.25	0.34	0.19	0.17	0.25	−0.71	0.51	1.00	−0.34	−0.07
P	−0.02	−0.18	0.35	0.15	0.17	0.49	0.62	0.70	0.79	0.82	0.20	−0.31	1.00	0.04
GY	−0.23	−0.02	−0.01	0.17	0.04	0.10	−0.07	0.00	−0.04	0.05	0.00	−0.08	0.01	1.00
